# Advancing clinical precision medicine via peripheral blood immune single‐cell omics

**DOI:** 10.1002/ctm2.70727

**Published:** 2026-06-22

**Authors:** Liyang Li, Jialu Hu, Wanxin Duan, Yan‐Ming Xu, Jiaqiang Zhang, Xiangdong Wang

**Affiliations:** ^1^ Department of Pulmonary and Critical Care Medicine, Zhongshan Hospital Fudan University Shanghai Medical College Shanghai China; ^2^ Department of Cardiology, Zhongshan Hospital Fudan University Shanghai Medical College Shanghai China; ^3^ Fudan University Center of Clinical Bioinformatics Shanghai Institute of Clinical Bioinformatics Shanghai China; ^4^ Shantou University Institute of Stereo‐Cell Biomedicine Shantou China; ^5^ Department of Anesthesiology and Perioperative Medicine, Center for Clinical Single Cell Biomedicine, Henan Provincial People's Hospital People's Hospital of Zhengzhou University Zhengzhou China

Single‐cell RNA sequencing (scRNA‐seq) has been applied for more than a decade to deeply investigate morphological and functional subtypes, subsets and substates of circulating and tissue resident cells under both physiological and pathophysiological conditions. With improvements in technological stability and reductions in economic burden, peripheral blood scRNA‐seq has been proposed as a routine measurement in clinical haematology and biochemistry to comprehensively understand systemic immune cell states, enable early detection of immune dysfunction, dynamically monitor disease progressions and therapeutic responses, and constantly mirror immune cell components and activities within the tissue microenvironment.^1^, ^2^, ^3^, ^4^, ^5^ One of the development milestones in clinical translation is the implementation of such high‐throughput scRNA‐seq at large scale in human populations across various ages, sexes, races, living conditions and disease states to establish standardised mean values and variations of each subtype, subset and substate of circulating immune cells under normal conditions. In addition, multiple dimensional, multi‐layered and functional single‐cell molecular profiles are increasingly critical for illustrating the complex interactions and heterogeneities among immune cells and for more closely visualising the complete biological image and regulations.

There is rapid increasing evidence to presenting a more comprehensive picture of systemic immune cell categories and functions through peripheral blood immune single‐cell multi‐omics (PBISM). Especially, it is important for clinical researchers and physicians to understand the implications and clinical value of PBISM‐generated data, multi‐dimensional molecular networks and disease‐specific features. PBISM integrated with DNA‐seq and scRNA‐seq has been applied to characterise genetic variants located close to genes that influence the corresponding mRNA abundance, statistically independent of other nearby cis‐expression quantitative trait loci (eQTLs) for the same gene, in a large‐scale cohort of Australian healthy individuals and patients with autoimmune diseases (e.g., nearly 1000 subjects, more than 1 million single cells and approximately 30 000 eQTLs).^6^ By integrating DNA‐seq and scRNA‐seq, more than 1.7 million polymorphic tandem repeats regulating gene expression and biological functions, and the significance of variation in blood cell‐type‐specific gene expression, were investigated in over 5.4 million blood immune cells from 1925 healthy individuals.^7^ This study defined the number of single‐cell expression quantitative trait tandem repeat loci, the specificity to immune cell types/subtypes, candidate causal drivers of gene expression, and genetic associations with immune‐mediated and haematological traits. Such population‐scale PBISM demonstrates the regulatory networks of cell type‐ and disease‐specific genes, provides new and deep insights into the genetic basis of human diseases, and discovers molecular drivers of individual heterogeneity in the immune system. These innovations on genetic changes in single‐cell and subtype levels can be the fountainhead for a new generation of clinical precision medicine.

The scope of comprehensive PBISM atlas is continuously expanding, and refreshing our knowledge about systemic immune function. A recently published Chinese Immune Multi‐Omics Atlas includes more than 10 million circulating immune cells from 428 Chinese adults, identifies 237 robust regulons, and reports 9600 eGenes and 52 361 caPeaks at cell‐type resolution.^8^ This is one of the important milestones in a PBISM‐based large‐scale studies of healthy populations and is one of the few PBISM human studies integrating scRNA‐seq with transposase‐accessible chromatin sequencing (scATAC‐seq), metabolomics, lipidomics, genome‐wide association study and functional phenomes. It is the first time to understand the immune cell subtype‐specific molecular correlations among gene regulatory networks, trans‐omic nodes, genetic mutations, cell type‐resolved QTL and phenomic characters. Single‐cell‐specific correlations between the epigenetic phenome and gene regulatory networks have been clarified in 10 million peripheral blood mononuclear cells across 1108 Finnish individuals by integrating the paired ATAC‐seq and RNA‐seq profiles in individual nuclei.^9^ The data from this particular study indicate that genetic variants exhibiting complete chromatin‐to‐expression cascades should be paid more attention in the prediction of disease relevance, and that the systematically weaker enhancer‒gene links play important roles in targeting disease‐variated genes. PBISM provides multi‐dimensional comprehensive profiles and spatiotemporal insights to guide clinical consideration and decision making, rather than relying on single gene mutations. The next generation of precision medicine can be individually developed on the basis of multi‐molecular targets, including gene expression and mutations, chromatin accessibility, enhancer‒gene links in cis or trans, gene modifications, metabolite networks and the cell‐type specificity and regulatory mechanisms of molecular QTLs in the single cells as well as single subtypes/subsets/substates. PBISM also demonstrates the molecular determinants of ultralong CAR T‐cell persistence and the roles of type 2 CAR T infusion products in the maintenance of leukaemia patient survival.^10^

In addition to the population size and measurement dimensionality, reference values of mean, median, minimum, maximum, standard error and standard deviation of each T‐cell subtype, subset and substate were calculated and presented to mimic the clinical biochemistry and haematology report. This was achieved after peripheral T‐cell scRNA‐seq in normal health and pre/post‐operative blood from in lung cancer patients was measured and the cell identity marker gene panels were evaluated and selected using the overlapped expression rate.^11^ This was the first attempt at a clinical scRNA‐seq report and the translational presentation of peripheral immune cell subtypes/subsets, although the trial needs to be furthermore validated and confirmed by a large scale of population. An Asian Immune Diversity Atlas as a multi‐national scRNA‐seq healthy reference atlas of human immune cells was systematically mapped and compared among various populations, including immune cell constructions and ratios, cell‐type and subtype identities, cell subtype/subset proportions and interactions in human diversities, and population‐specific and context‐dependent eQTL effects.^12^

The next step towards clinical precision medicine is to distill comprehensive, multi‐dimensional PBISM‐derived information and translate it into clinical practice. One of the challenges hindering the clinical translation of scRNA‐seq is the need to standardise the definitions of single‐cell identity marker gene panels (ciMGPs) and to clarify the specificities of their representatives for cell categories and functions.^13^ Persistent questions include where those ciMGPs come from, why those genes are selected into the panel, how the number of genes is decided, and whether such concept of single‐cell identity can be readily accepted by clinicians (Figure 1A). Recently, a new discipline coined ‘stereological cell biomedicine’ was considered as a milestone to improve the application and to accelerate the translation of scRNA‐seq and as a new source and hope to create the new generation of clinical precision medicine.^14^, ^15^, ^16^ StereoCell‐seq, one of the breakthrough technologies, mainly profiles transcriptomic and/or multi‐omic data of an intact cell isolated from tissues, blood, bioliquids or biopsies, whose cell identity has been pre‐validated via cell morphology and surface biomarkers.^17^ Although multi‐omics of intact cells are measured by a spatial enhanced‐resolution single‐cell sequencing platform based on high‐density DNA nanoball‐patterned arrays, the resulting data are presented in a two‐dimensional pattern (Figure 1B). The stereological profiles of PBISM should be further illustrated using the spatiotemporal cell surfaceomics, multi‐dimensional molecular networks, virtual cell models and organelle‐level interactions (Figure 1C–1E).

**FIGURE 1:**
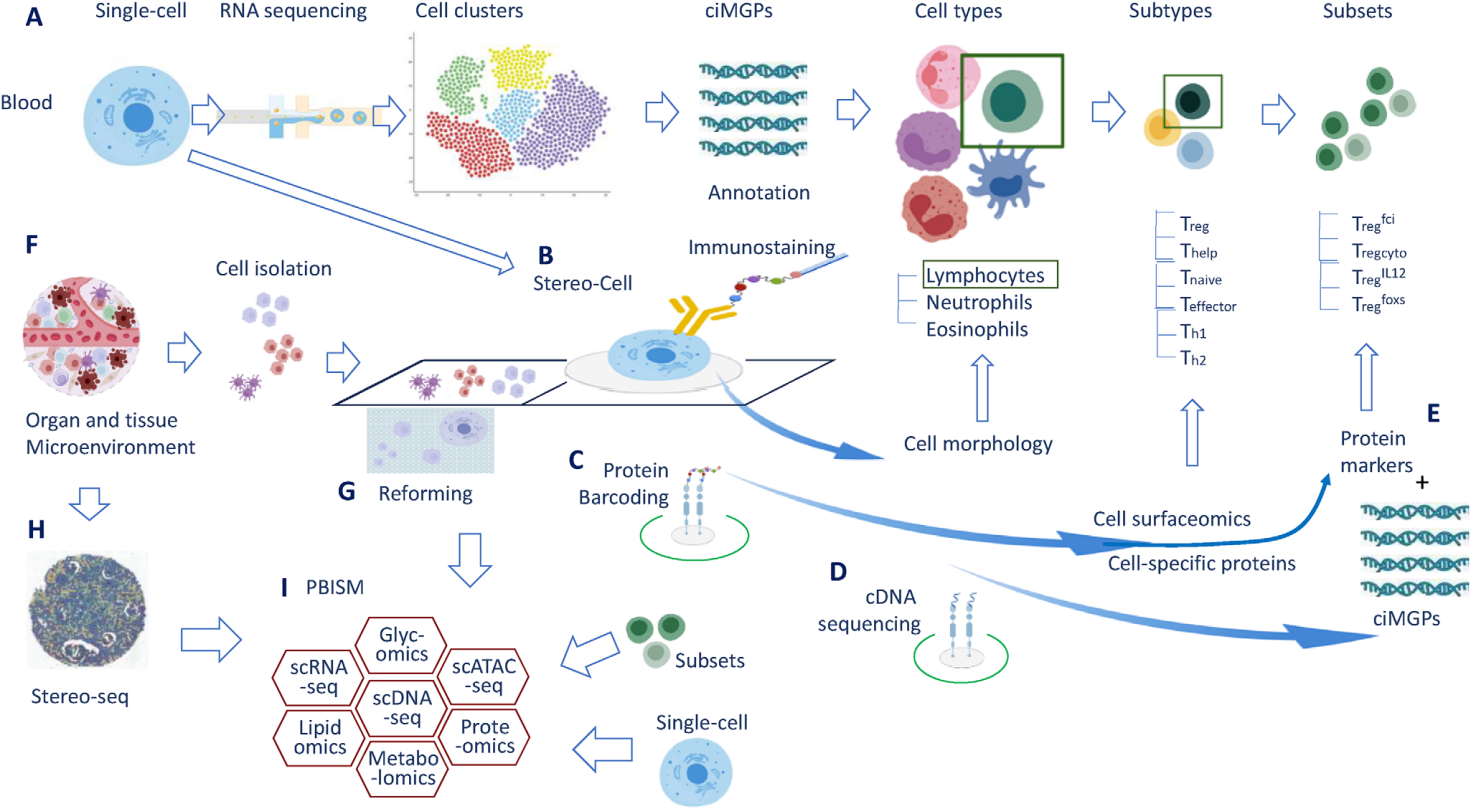
Summary of comprehensive single‐cell measurement workflow. (A) Overview of peripheral blood immune cell isolation for single‐cell RNA sequencing (scRNA‐seq) and cell cluster detections. The identities of cell types, subtypes and subsets (or/and functional substates) in various clusters are annotated using cell identity marker gene panels (ciMGPs). (B) Concept of Stereo‐cell coined as a spatial enhanced‐resolution single‐cell sequencing platform based on high‐density DNA nanoball‐patterned arrays to capture cells at the high‐fidelity transcriptome profiling. An intact cell lied on the plate can be stained with haematoxylin eosin dye and fluorescence to detect cell morphology, and/or immunoassayed with cell‐type and subtype‐specific biomarkers using antibodies, before performing scRNA‐seq. (C) The cell is labelled with digital barcodes and sequenced to detect a large number of cell surface proteins coined as the surfaceomics before scRNA‐seq. The cell‐type‐specific profiles of surfaceomics present the cell biological functions and define the precise categories. (D) The transcriptomic profiles of intact cell are measured using scRNA‐seq after the cell is identified. (E) The cell surfaceomics are integrated with ciMGPs after Stereo‐cell‐seq to furthermore categorise cell subsets, of which the accuracy can be compared and/or matched with those from scRNA‐seq alone. (F) The peripheral immune cells are traced and uncovered in the microenvironment of normal and pathological tissues. (G) Those tissue cells are isolated and seeded on Stereo‐cell plates and then applied for the investigation of molecular mechanisms by which those cells are reorganised, reformed, and remodelled into the microenvironment under the regulation of peripheral immune cells. (H) The tissue is also prepared for histology, immunostaining, in situ hybridisation and spatial enhanced resolution omics sequencing (Stereo‐seq). (I) The multiple molecular profiles of peripheral immune cells (PBISM) from blood or tissues after scRNA‐seq‐based ciMGPs or Stereo‐cell‐seq‐based multiple labels are measured, including transcriptomic regulatory networks (scRNA‐seq), glycan repertoires (glycomics), transposase‐accessible chromatins (ATAC‐seq), structure and function of the lipid elements (lipidomics), exact sequence of nucleotides in a DNA molecule (scDNA‐seq), the entire set of proteins (proteomics), as well as networks and interactions between metabolites (metabolomics).

Single‐cell biomedicine is entering an exciting moment due to the rapid increase in the large scale of human population‐based PBISM studies, the dimensions of intracellular molecular regulatory networks, the layers of various multi‐omic profiles and the interorganellar communication. The PBISM is a powerful source for generating multi‐dimensional information for the establishment of clinical artificial intelligent cells, digital cells and virtual cells, which are dependent upon the matching degrees of multi‐omic profiles. The single‐cell models and values of biology, diagnosis, monitoring and prediction for the disease and therapy are highly dependent upon the consistency with biological and clinical phenomes. Complementarities between small‐ and large‐scale human studies, an intact cell by StereoCell‐seq and tissue Stereo cells by continuous Stereo‐seq, and gene expression and metabolites will provide more potential and precise insights into disease occurrence and treatment (Figure 1F–1G). PBISM represents an important approach to spatiotemporally exhibit cell lifestyle, biological behaviour, living microenvironment and interacting factors (Figire 1F), although the rationality and practicality of PBISM for clinical practice need to be validated. PBISM enables access to the actual complexity of the cell, deepens the molecular mechanism of organellar interactions, and translates the comprehensive understanding into the designs and strategies of clinical precise medicine. The strategic impact of those large‐scale human PBISM studies lies in the creation of a panoramic cell data framework in the form of a ‘digital twin‐cell’. PBISM is expected to ensure representativeness with high quality and sufficient scale, the combination of quality and quantity, the dynamic promotion with the fast development of biotechnologies, and guarantee continuity of performance and data safety.

The significance of PBISM is to revolutionarily refresh our knowledge and understanding of clinical precision medicine and provide a new concept and approach to design the diagnostic and therapeutic strategy. PBISM presents clinicians a system immunity of the disease at the single‐cell solution and with multi‐modal molecular depth, and transform the precision medicine from static, tissue‐limited and two‐dimensional characterisations to dynamic, holistic and multi‐dimensional immune monitoring and decision making. PBISM is a scientific and technical innovation in precision medicine and a clinical and theoretic breakthrough in single‐cell biomedicine. New therapeutic strategies for precision medicine can be designed on the basis of dynamical immune cell heterogeneity and monitored by a multi‐dimensional, high‐resolution and non‐invasive perspective for clinical practice (Figure 1I). Designing precision medicine with PBISM requires moving from the discovery and development phase to the clinical validation mindset, establishing a closed‐loop system from blood sampling, multi‐omic measurement, artificial intelligent analysis and interpretable report, to guided clinical action, outcome tracking and refined algorithm at key clinical decision points. PBISM is an accessible and powerful potential with far‐reaching impacts, promoting a shift in patient management from reactive, trial‐and‐error model to a proactive, dynamically adaptive and deeply personalised science. PBISM‐based precision medicine can be designed with clear targeting of disease mechanisms, monitored by specific biomarkers, performed under clinical model construction, detailed by individualised interventions, and applied with dynamic efficacies in real‐world clinical settings.

In conclusion, immune single‐cell multi‐omics is a critical tool to deeply understand the systemic immune environment, peripheral cell‒cell communication and immune reflections of the tissue microenvironmental immunity. Large‐scale population cohorts of PBISM become a key infrastructure connecting single‐cell biomedicine with medical practice. By integrating multidimensional and high‐quality data of PBISM, cohort studies are important to validate the real‐world applicability of single‐cell measurement and to develop new generations of clinical precision medicine for disease prevention and medical decision making. There are urgent needs for well‐designed and rigorously constructed large‐scale PBISM population cohorts, sustained long‐term funding support, robust data standardisation and security frameworks, and science‐based sharing mechanisms. Thus, the next steps of PBISM should advance into a stereological world of multi‐omic profiles, headline the spatiotemporal interactions among molecular regulatory networks and organellar communication, and provide solid and reliable evidence to support revolutionary innovations in clinical precision medicine.

## CONFLICT OF INTEREST STATEMENT

None of the authors have a conflict of interest to disclose.

## Data Availability

Data sharing not applicable to this article as no datasets were generated or analyzed during the current study.
